# pedigreejs: a web-based graphical pedigree editor

**DOI:** 10.1093/bioinformatics/btx705

**Published:** 2017-10-31

**Authors:** Tim Carver, Alex P Cunningham, Chantal Babb de Villiers, Andrew Lee, Simon Hartley, Marc Tischkowitz, Fiona M Walter, Douglas F Easton, Antonis C Antoniou

**Affiliations:** 1 Centre for Cancer Genetic Epidemiology, Department of Public Health and Primary Care, Strangeways Research Laboratory, University of Cambridge, Cambridge, UK; 2 The Primary Care Unit, Department of Public Health and Primary Care, University of Cambridge, Cambridge, UK; 3 Department of Medical Genetics and National Institute for Health Research Cambridge Biomedical Research Centre, University of Cambridge, Cambridge, UK; 4 Centre for Cancer Genetic Epidemiology, Department of Oncology, University of Cambridge, Cambridge, UK

## Abstract

**Motivation:**

The collection, management and visualization of clinical pedigree (family history) data is a core activity in clinical genetics centres. However, clinical pedigree datasets can be difficult to manage, as they are time consuming to capture, and can be difficult to build, manipulate and visualize graphically. Several standalone graphical pedigree editors and drawing applications exist but there are no freely available lightweight graphical pedigree editors that can be easily configured and incorporated into web applications.

**Results:**

We developed ‘pedigreejs’, an interactive graphical pedigree editor written in JavaScript, which uses standard pedigree nomenclature. Pedigreejs provides an easily configurable, extensible and lightweight pedigree editor. It makes use of an open-source Javascript library to define a hierarchical layout and to produce images in scalable vector graphics (SVG) format that can be viewed and edited in web browsers.

**Availability and implementation:**

The software is freely available under GPL licence (https://ccge-boadicea.github.io/pedigreejs/).

**Supplementary information:**

Supplementary data are available at *Bioinformatics* online.

## 1 Introduction

The collection, management and visualization of pedigree (family history) data is a core activity in clinical settings, such as genetic centres and primary care, where healthcare professionals manage patients at risk of disease. Pedigree data are used to identify individuals at risk of familial disease and to inform their clinical management, including referral of individuals at elevated risk for mutation screening and/or recommending enhanced screening. For example, generating a pedigree is a requirement for calculating *BRCA1* and *BRCA2* mutation carrier probabilities and breast cancer risks in the BOADICEA (Lee *et al.*, 2014) and IBIS (Tyrer *et al.*, 2004) programs recommended in the National Institute for Health and Care Excellence guidelines (NICE, 2013, https://www.nice.org.uk/guidance/cg164).

Clinical pedigree datasets can be difficult to manage for several reasons: (i) they are supplied by humans not machines, and are therefore time consuming to capture in digital form; (ii) they include both family structure (which can be difficult to visualize in the presence of complex structure and still remain clear and comprehensible) and separate information for each family member (which must be linked); (iii) they often require updating over time; (iv) they can include data errors (e.g. broken family structures) and internal inconsistencies; and (v) they must be stored securely in order to fulfill data privacy requirements.

**Fig. 1:**
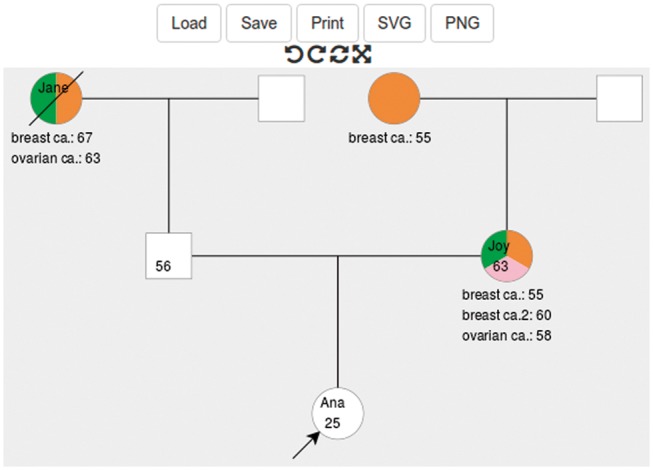
Screenshot showing a pedigree extending to second degree built using pedigreejs. The pedigree is annotated in the conventional manner: the proband is identified with an arrow; family members who have developed cancer are shaded; and unaffected family members are unshaded. Users can add or delete family members, and change their properties using widgets, which appear when the individual is selected. Buttons directly above the pedigree are used to ‘undo’ and ‘redo’ actions, ‘reset’ the initial structure, and switch to ‘fullscreen’ mode. The buttons at the top are used to ‘Load’ and ‘Save’ pedigree data files, to print the pedigree, and to export it as a SVG/PNG image

Many existing software applications capture and display pedigree data. Some open-source tools (e.g. Kinship2: Sinnwell *et al.*, 2014) generate a vector image file from an input data file, whereas others (e.g. Pelican: Dudbridge *et al.*, 2004 and PhenoTips: Girdea *et al.*, 2013) provide a graphical pedigree editor. There are also commercial subscription pedigree drawing services (http://www.progenygenetics.com, http://pedigreedraw.com/, http://www.pedigreexp.com). However, after evaluating available tools (see Supplementary Data) we found no freely available lightweight pedigree drawing tools (components implemented solely in JavaScript) that could be easily incorporated into other web applications, and configured to provide a means of capturing and visualising pedigree data. Pedigreejs has been developed to fulfil these requirements as part of the ongoing BOADICEA project (http://ccge.medschl.cam.ac.uk/boadicea/, Lee *et al.*, 2014) (Fig. 1).

## 2 Materials and methods

Pedigreejs has been implemented as a configurable JavaScript library to provide a graphical pedigree editor in a web-based environment. The pedigreejs algorithm uses the d3-hierarchy JavaScript module (https://d3js.org) to assist the layout of individuals’ nodes in the pedigree representation. Pedigrees are rendered as SVG images in the browser window which results in a fast and responsive user experience to facilitate clinical data entry. The standard nomenclature for representing pedigrees (Bennett *et al.*, 1995, 2008) is used. Pedigrees are built by adding parents, partners and siblings (including twins) using widgets that appear on mouseover of an individual.

Local browser storage is used to store the pedigree data (including family structure and linked data for each family member, e.g. sex, age). This means that confidential patient data can be stored on the client computer and not transmitted over the Internet. For reasons of data privacy the default is to hold these data for the session so that they do not persist after the browser or tab is closed. Previous versions of the pedigree dataset are also stored so that when a change is made it can be undone. Basic validation of the dataset is provided by default (e.g. check all mothers are female) and this can be overridden to define specific validations.

The pedigree data are stored in the lightweight JSON format (https://www.w3schools.com/js/js_json_intro.asp) to facilitate data interchange with other applications. JSON is easily extendable, so that the individual level data that are gathered are not limited. Once constructed, the pedigree dataset can be saved to a JSON format data file and loaded back into pedigreejs at a later date, so that pedigrees can be easily updated. In addition, pedigree data files in BOADICEA Web Application (v4) pedigree format and PED format (used by PLINK http://www.cog-genomics.org/plink/1.9, Chang *et al.*, 2015) can be loaded into pedigreejs.

Pedigreejs has a variety of configuration options to assist integration with other web applications. For example, it can be configured to define the size of the SVG image that is rendered on the web-page, and define whether the image can zoom in and out. To allow for different property editors, an edit function can be provided in the configuration. In addition diseases can be configured and colours defined to identify affected individuals.

## 3 Results

Pedigreejs is a freely available, configurable, interactive, fast and responsive JavaScript pedigree editor. It is lightweight (implemented solely in JavaScript) and uses D3.js to assist in the rendering and does not send requests to a backend server. Creating and deleting individuals is made easy by clicking on widgets that appear on mouseover of a family member. ‘Undo’, ‘redo’ and ‘reset’ options and the ‘fullscreen’ option add to this being a user friendly editing tool.

Future developments will include further support for other commonly used pedigree data file formats (e.g. Madeline, Progeny). Pedigreejs will also be updated to support more complex family structures including consanguineous partners beyond those at the same level in the tree such as cousins. Pedigreejs will be used in future releases of the BOADICEA Web Application.

## Supplementary Material

Supplementary Data
