# Supplementary material for: pedigreejs: a web-based graphical pedigree editor
Source: Bioinformatics. 2017 Oct 31;34(6):1069–71. doi: 10.1093/bioinformatics/btx705 (PMC5860312; doi:10.1093/bioinformatics/btx705)
Supplement: Supplementary Data [file btx705_supplementary_resubmit.docx]

1. **Table 1. Comparison of open-source graphical pedigree tools**

|  | 1. **Pedigree Tools** | | | | | |
| --- | --- | --- | --- | --- | --- | --- |
|  | 1. **Cranefoot** | 1. **Pelican** | 1. **Kinship2** | 1. **Madeline 2.0** | 1. **HaploPainter** | 1. **pedigreejs** |
| **Interface** | 1. Command line | 1. Java Swing | 1. R | 1. Command line | 1. Perl/Tk | 1. Web-browser |
| **First release** | 2005 | 2004 | 2015 | 2007 | 2004 | 2017 |
| **Last update** | 2009 | 2014 | 2015 | 2016 | 2008 | 2017 |
| **Interactive Visualisation** | ✘ | ✓ | ✘ | ✓ | ✓ | ✓ |
| **Configurable Annotations** | ✓ | ✘ | ✓ | ✓ | ✓ | ✓ |
| **Consanguineous relationships** | ✓ | ✓ | ✓ | ✓ | ✓ | ✓ |
| **Graphical Output** | Postscript | PNG,  JPEG,  Postscript | PNG | SVG | SVG, Postscript, PDF, PNG | Postscript, PNG, SVG |
| **Supported Formats** | Linkage | Linkage, SLINK |  | Madeline,  Madeline-XML | linkage, HaploPainter, SQL | BOADICEA (v4),  PED |
| **URL** | www.finndiane.fi/software/cranefoot/ | sites.google.com/site/fdudbridge/software/pelican | cran.r-project.org/web/packages/kinship2 | madeline.med.umich.edu/madeline/ | haplopainter.sourceforge.net | ccge-boadicea.github.io/pedigreejs/ |
